# 3D-Printed Composite Scaffolds Containing SiO_2_ for Bone Regeneration in *In Vivo* Models: A Systematic Review

**DOI:** 10.1021/acsomega.5c10447

**Published:** 2026-05-14

**Authors:** Giovanna do Espirito Santo, Marcelo Assis, Homero Garcia-Motta, Karolyne dos Santos Jorge Sousa, Amanda de Souza, Mariana Carvalho Simões, Flávia de Oliveira, Daniel Ribeiro, Ana Claudia Muniz Rennó

**Affiliations:** † Department of Biosciences, 28105 Federal University of São Paulo (UNIFESP), Silva Jardim Street, 136, Santos, SP 11015020, Brazil; ‡ Federal University of São Carlos (UFSCar), Washington Luís Highway, km 235 - SP-310, Sao Carlos, SP 13565905, Brazil

## Abstract

Bone fractures are a growing global burden due to population aging and increasing osteoporosis incidence, leading to impaired healing and a demand for advanced regenerative strategies. Three-dimensional (3D) printed scaffolds have emerged as promising biomaterial platforms owing to their ability to mimic native bone architecture and deliver bioactive cues. Among the bioactive components explored, silica (SiO_2_) has been shown to modulate osteoblast proliferation, promote angiogenesis, and enhance mineralized matrix deposition when incorporated into calcium- and phosphate-containing biomaterials. This systematic review aimed to evaluate 3D-printed composite scaffolds containing SiO_2_ used in *in vivo* bone defect models, focusing on their biological performance and regenerative outcomes based on studies retrieved from PubMed, Scopus, Embase, and Web of Science published between 2005 and 2025. It is important to emphasize that this review focuses on composite biomaterial systems in which SiO_2_ is incorporated as a bioactive component, rather than on structurally pure SiO_2_-containing composite scaffolds. The included reports demonstrated that both synthetic and biogenic SiO_2_ modulate key scaffold characteristics, including porosity, surface topography, wettability, and degradation behavior. Architectures with interconnected pores ranging from 200 to 600 μm, increased surface roughness, and improved hydrophilicity facilitated protein adsorption and cell adhesion, while variations in particle morphology and crystallinity influenced ion release kinetics and osteogenic signaling. *In vivo* studies conducted mainly in rodent and rabbit critical-sized defect models consistently showed enhanced bone formation in SiO_2_-containing scaffolds, as demonstrated by Micro-CT analysis, histomorphometry, and the expression of osteogenic markers. Additionally, SiO_2_-containing scaffolds were shown to modulate local inflammatory responses and upregulate osteogenic gene expression, particularly when combined with calcium phosphate phases. Overall, SiO_2_-functionalized 3D-printed scaffolds represent a promising strategy for bone tissue engineering when combined with calcium phosphate phases, due to their synergistic effects on surface bioactivity, osteogenic signaling, and bone regeneration.

## Graphical abstract

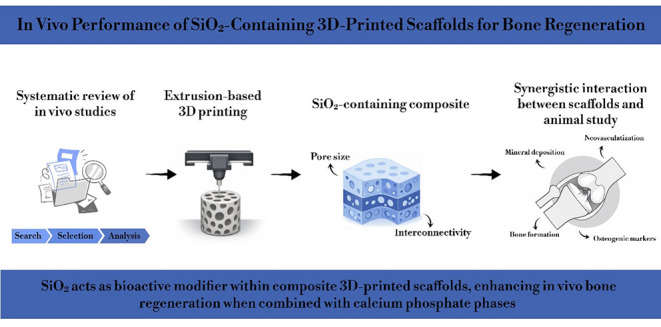

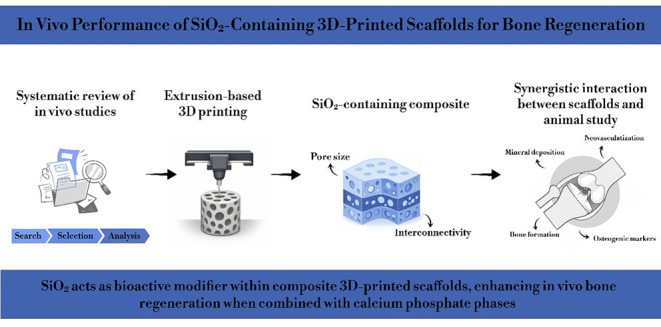

## Introduction

In recent years, the development of biomaterials has gained attention, particularly in response to the rising life expectancy and the growing need for innovative medical solutions to treat age-related conditions. Among these challenges, bone fractures and defects represent a significant concern, as the aging population is more susceptible to osteoporosis and delayed bone regeneration.^1^ In this context, biomaterials designed to enhance bone repair play a crucial role, with scaffolds emerging as promising structures for guiding tissue regeneration. Specifically, scaffolds incorporating silica (SiO_2_) have attracted interest due to their ability to stimulate bone healing, making them a valuable approach in bone tissue engineering.^2^

SiO_2_ is a chemical compound composed of silicon and oxygen atoms and has been extensively studied for its applications in cements, glasses, and composite materials.^1,2^ This material stands out for its remarkable thermal stability, chemical resistance, and ability to adapt to different structural forms.^3,4^ In addition, silicon-containing materials exhibit significant biological potential and bioactivity by interacting with physiological fluids, promoting the formation of surface silanol (Si–OH) groups. Due to the partial negative charge of the Si–OH groups, adsorption of Ca^2+^ ions occurs, forming a Ca^2+^-rich surface layer. This layer then attracts phosphate ions, initially giving rise to amorphous calcium phosphate, which evolves into the formation of carbonated hydroxyapatite, similar to the natural mineral component of bones.^3^ In this way, SiO_2_ acts as a bioactive nucleating substrate, not merely an inert support. This apatite layer facilitates cellular adhesion, enhances scaffold integration with surrounding bone tissue, and promotes osteoconductivity, improving the overall regenerative process.^5,6^ Wu et al. developed porous SiO_2_-containing composite scaffolds inspired by mussel adhesion, demonstrating that these structures enhance apatite mineralization and promote the attachment and proliferation of bone marrow stromal cells (BMSCs), which are essential for bone regeneration due to their ability to differentiate into osteoblasts.^7^ Similar findings were reported by Kavya et al.,^8^ who demonstrated that nano-SiO_2_ particles grafted onto carbon fiber surfaces, using a self-assembly strategy to strengthen the interfacial bonding in chitosan/gelatin/SiO_2_ composite scaffolds, exhibited no cytotoxic effects on MG63 cell adhesion. Beyond experimental and preclinical investigations, SiO_2_-based materials have been successfully translated into clinical practice for bone repair, particularly in the form of bioactive glasses. These materials, typically composed of a SiO_2_-rich network modified with oxides such as CaO, Na_2_O, and P_2_O_5_, are widely recognized for their ability to bond directly to living bone through the formation of a biologically active hydroxycarbonate apatite layer at the material–tissue interface.^8,9^ Clinically approved bioactive glass devices, including S53P4 (BoneAlive), NovaBone, and Cortoss, have been employed as bone graft substitutes in orthopedic, spinal, and maxillofacial applications, demonstrating effective osteointegration, bone regeneration, and long-term clinical stability.^9–11^ The clinical success of these SiO_2_-based materials is attributed to their surface reactivity in physiological environments, which leads to ionic exchange, silica gel layer formation, and subsequent apatite nucleation, closely mimicking the mineral phase of natural bone.^8,12^ Furthermore, the controlled release of soluble silicon species from these materials has been associated with enhanced osteoblast proliferation, differentiation, and angiogenic responses, contributing to improved bone healing outcomes in clinical and translational studies.^13,14^ These findings highlight the relevance of SiO_2_ not only as a structural component in experimental scaffolds but also as a key bioactive phase in clinically validated medical devices for bone repair. Furthermore, the controlled release of soluble silicon species from these materials has been associated with enhanced osteoblast proliferation, differentiation, and angiogenic responses, contributing to improved bone healing outcomes in clinical and translational studies.^13,14^ These findings highlight the relevance of SiO_2_ not only as a structural component in experimental scaffolds but also as a key bioactive phase in clinically validated medical devices for bone repair.

Despite the promising results of modified SiO_2_-based systems, such as ion-doped or composite formulations, pure SiO_2_ exhibits limited intrinsic bioactivity, and its effective application in bone tissue engineering relies on compositional and structural modifications to enhance biological performance.^10^ In this context, 3D printed Si-based scaffolds have gained significant attention.^10^ 3D printing offers several advantages for biomaterials, including precise control over scaffold architecture, reduced production time and costs, and the ability to create biocompatible structures that integrate seamlessly with bone tissue.^14^ This technology enables the fabrication of scaffolds that closely replicate the natural porous structure of bone, promoting cell adhesion and providing a conducive environment for new bone formation.^15,16^

The use of 3D-printed SiO_2_-based composite scaffolds holds significant therapeutic potential; however, important knowledge gaps still limit clinical translation. Although additive manufacturing and biomaterials science have advanced considerably, *in vivo* studies frequently lack methodological standardization, resulting in variability in biomechanical performance, degradation behavior, and osteointegration outcomes. Moreover, long-term effects, tissue interactions, and large-scale applicability remain insufficiently investigated. This review systematically analyzes available *in vivo* evidence, providing a structured evaluation of therapeutic potential, biocompatibility, and functional performance. By identifying current limitations and research priorities, this study contributes to the optimization and translational advancement of silica-containing composite scaffolds.

## Material and Methods

### Search and Criteria for Selecting Articles

The systematic review methodology followed the guidelines outlined in the Cochrane manual and the PRISMA declarations.

### Eligibility Criteria

#### Types of Studies

Studies evaluating 3D-printed composite scaffolds containing SiO_2_ in *in vivo* bone defect models were included. The searched articles were limited to articles published in English, including with publication date ranging from January 2005 to September 2025. Review articles, as well as gray literature were summarily excluded. Moreover, comprehensive exploration encompassing the physical-chemical characterization of the coating, alloy and stainless steel, *in vitro* studies, *in situ* investigations, reviews, case reports, and book chapters, studies without methodological description and results were excluded.

#### Types of Interventions

This review included *in vivo* studies involving the implantation of 3D printed SiO_2_-containing composite scaffolds for bone defect repair and bone regeneration, focusing on their structural performance, osteointegration, biodegradability, and therapeutic efficacy in promoting bone regeneration.

#### Types of Results

To address the central question of this study, results from studies focusing on histological and histomorphometric parameters were analyzed. These analyses included observations obtained through optical microscopy, scanning electron microscopy, and computerized microtomography. Additionally, data related to other *in vivo* analysis parameters were extracted from the included articles. The evaluation also considered whether comparisons were made with or without biomaterial implantation.

The primary variable was based on histological analysis. Furthermore, the following variables were extracted: authors, species/strain, animal sex, age, weight, type and size of defect, implantation periods, physicochemical characterization methods, *in vivo* analyses, results, and outcomes.

Quantitative synthesis or meta-analysis was not performed due to substantial heterogeneity among the included studies, including differences in animal species, defect size and anatomical location, scaffold composition, implantation periods, and outcome assessment methods. Therefore, qualitative synthesis was considered the most appropriate approach.

### Information Sources

The information databases of Embase (https://www.embase.com) PubMed (https://pubmed.ncbi.nlm.nih.gov/), Scopus (https://www.scopus.com/), and Web of Science (https://www.webofscience.com/) were used as a data source for the survey of publications.

### Search Strategy

The search strategy was developed according to PRISMA guidelines and applied to Embase, PubMed, Scopus, and Web of Science. The following reference search string was used and adapted to the syntax of each database: (“silica” OR “SiO_2_”) AND (“scaffold” OR “3D-printed scaffold”) AND (“*in vivo*”). The term “drug delivery” was excluded to avoid nonrelevant applications. Searches were limited to articles published in English between January 2005 and September 2025. Titles, abstracts, and keywords were screened.

### Selection of Articles and Application of Inclusion and Exclusion Criteria

Two reviewers (GES and MA) independently screened titles and abstracts according to the predefined exclusion criteria. Potentially eligible studies underwent full-text assessment to confirm compliance with inclusion criteria. Disagreements were resolved through discussion. Data extraction was performed independently using predefined variables, and discrepancies were addressed through structured consensus.

## Results

Figure 1 presents the PRISMA flow diagram illustrating the study selection process. A total of 929 records were identified from four databases (Embase, PubMed, Scopus, and Web of Science). After the removal of 267 duplicate records, 662 studies were screened based on their titles, resulting in the exclusion of 445 articles that did not address bone regeneration, SiO_2_-based materials, or 3D-printed scaffolds. Subsequently, 217 articles were evaluated by abstract screening, and 152 studies were excluded due to the absence of *in vivo* experiments, lack of bone defect evaluation, or insufficient methodological information. Following full-text assessment, 56 articles were excluded because of inappropriate experimental design, use of nonrelevant animal models, or absence of 3D printing. Ultimately, nine studies met all eligibility criteria and were included in this systematic review.

**Figure 1:**
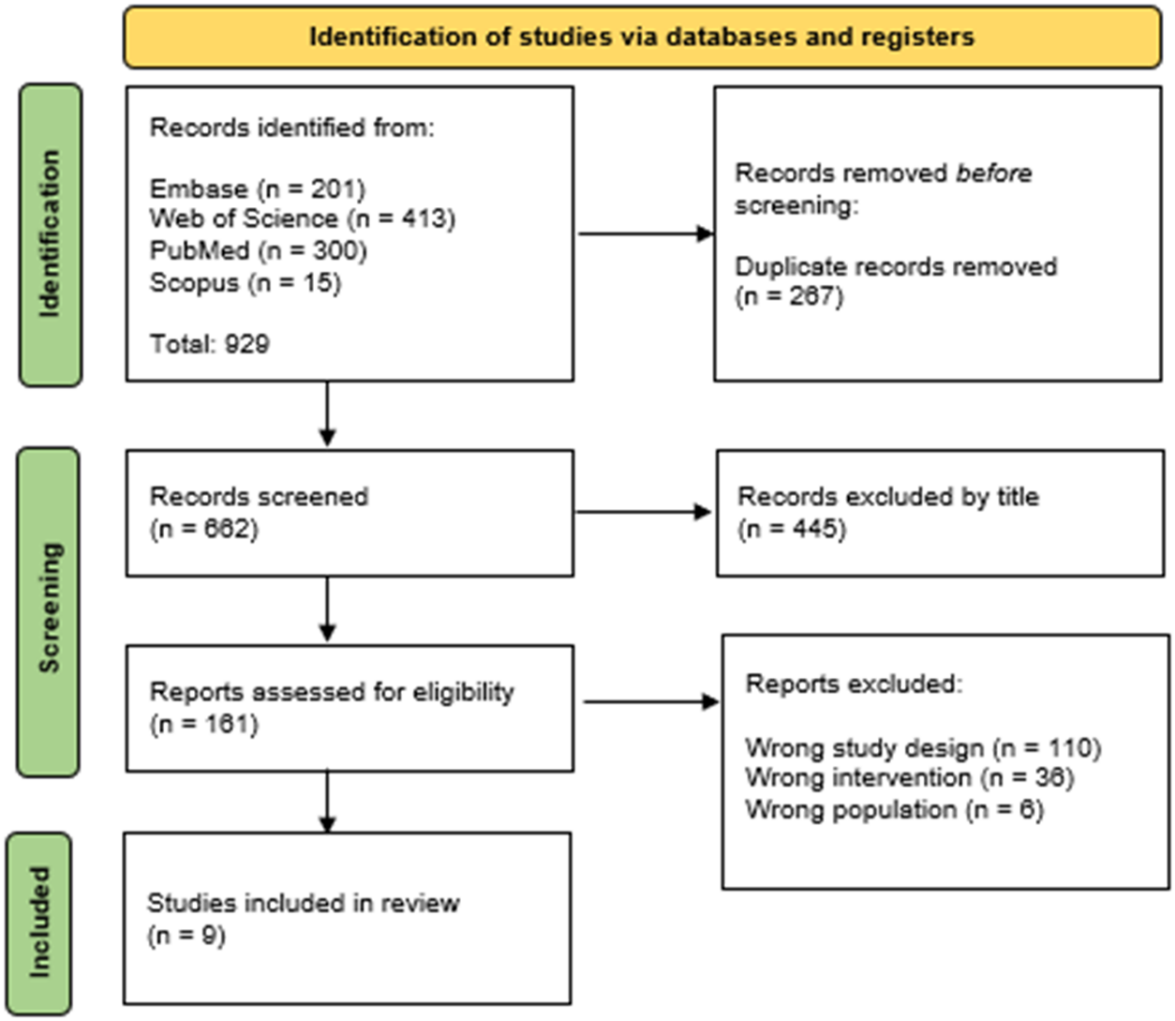
PRISMA flow diagram of search strategy.

Table 1 summarizes the characteristics of the included studies, detailing the animal species, age, weight, defect type, size, and implantation period. In terms of animal species, 2 studies used Wistar rats,^17,18^ while three studies utilized Sprague–Dawley rats.^19–21^ Another study used rats, though the strain was not specified.^22^ Additionally, three studies employed New Zealand rabbits.^23–25^ For animal sex, six studies used male animals^17,19–21,26^ one study used female animals,^18^ and two studies have not specified the gender.^22,23^

**Table 1:** Overview of Animals Used in *In Vivo*

**authors**	**species/Strain**	**age**	**weight (kg)**	**defect type**	**size of defect**	**implantation periods**
Abdian et al., 2024	Rats (Male)	N.D.	250 g	Calvarial/Cranial defect model	5 mm defect on both sides of the skull	3 weeks
Sousa et al., 2023	Wistar rats (Male)	12 weeks old	300–350 g	Calvarial/Cranial defect model	8 mm critical bone defect	15 and 45 days
Knabe et al. 2023	Wistar rats (female)	Six months old	Approximately 354g	Femoral segmental defect model	6 mm critical size segmental defect	3 and 6 months
Yuan et al., 2022	New Zealand white rabbits	Adult	N.D.	Femoral trochlear cartilage defect model	4 mm × 1 mm cylinder defect	1 and 3 months
Liu et al., 2020	Sprague–Dawley rats (male)	N.D.	200–250 g	Calvarial/Cranial defect model	8 mm diameter	2, 4, 8, and 12 weeks
Lee et al., 2020	New Zealand rabbits (male)	6 months old	2.5–3.5 kg	Midshaft of the radius defect model	15 mm	4, 8, and 12 weeks.
Ke et al., 2019	Rat	N.D.	300 g	Distal femoral model	3.4 mm × 5.2 mm	8, 12, and 16 weeks.
Li et al., 2017	New Zealand rabbits (male)	6 months old	2.5–3.5 kg	Midshaft of the radius defect model	15 mm	4, 8, and 12 weeks
Naga et al., 2014	Sprague–Dawley Rats (male)	Adults	200– 250 g	Femoral diaphysis region defect model	2 mm in diameter	5 months

Regarding age, three studies used 6-month-old rabbits,^18,24,25^ one used 12-week-old rats,^17^ and two included adult animals without specifying an exact^21,23^ The remaining studies did not report age.^19,20,22^ The Wistar rats weighed between 300 and 354 g,^17,18^ while the Sprague–Dawley rats ranged from 200 to 250 g.^19–21^ The rabbits weighed between 2.5 and 3.5 kg,^24,25^ except in one study where weight was not specified.^23^

Different defect models were used across the studies. Among the Wistar rats, two studies applied a calvarial/cranial defect model with sizes between 5 mm and 8 mm,^17,19^ and 1 created a femoral segmental defect of 6 mm.^18^ Among the Sprague–Dawley rats, two studies also used cranial defects of 8 mm^20^ or femoral diaphysis defects of 2 mm.^21^ Other models included a distal femoral defect of 3.4 mm × 5.2 mm in unspecified rats^22^ and trochlear cartilage or midshaft radius defects in rabbits.^23–25^ Implantation periods ranged from 2 weeks to 6 months.^18,20^ Most studies analyzed multiple time points, varying between 3 weeks and 5 months.^17,19,21,22^ For midshaft radius defects, the evaluation periods were 4, 8, and 12 weeks.^24,25^ Studies on cranial defects also included assessments up to 12 weeks.^20^

In Table 2, the characteristics of the SiO_2_ particles obtained in the articles reviewed are shown, highlighting their physicochemical properties and synthesis methods. Related to the raw material used for the 3D printed scaffolds most of the authors used SiO_2_ combined with other materials. Three studies used β-TCP combined with SiO_2_,^18,22,25^ while two incorporated alginates.^17,24^ Additionally, two studies used chitosan-based composites,^19,23^ and one study included gelatin in combination with alginate and SiO_2_.^20^ A single study incorporated multiple oxides, including Al_2_O_3_, SiO_2_, and TiO_2_.^21^

**Table 2:** Physicochemical Properties and Synthesis Methods^a^

**authors**	**material**	**synthesis of SiO**_**2**_	**morphology**	**size**
Abdian et al., 2024	Chitosan/hydroxyapatite/SiO_2_	Stöber method	Spheres	200 nm
Sousa et al., 2023	Alginate/SiO_2_	From marine sponge *Dragmacidon reticulatum*	Spicules	>30 μm
Knabe et al. 2023	β-TCP/SiO_2_	Commercial	-	-
Yuan et al., 2022	Chitosan/SiO_2_	Stöber method	Spheres	74 nm
Liu et al., 2020	Gelatin/Alginate/SiO_2_/of rat bone marrow mesenchymal stem cells	Laponite nanosilicate	Needles	>50 nm
Lee et al., 2020	Alginate/SiO_2_	LUDOX TM-50	Spheres	45 nm
Ke et al., 2019	β-TCP/SiO_2_	Commercial	-	-
Li et al., 2017	β-TCP/SiO_2_	Stöber method	-	6.8 nm
Naga et al., 2014	Al_2_O_3_/SiO_2_/TiO_2_	Stöber method	Spheres	-

a Legend: Al_2_O_3_: Aluminum Oxide, β-TCP: β-Tricalcium Phosphate, SiO_2_: Silicon Dioxide, TiO_2_: Titanium Dioxide.

Regarding obtaining these particles, four studies used the Stöber method for SiO_2_-based material synthesis,^19,21,23,25^ while three relied on commercially available materials.^18,22,24^ One study obtained SiO_2_ from a marine sponge,^17^ and another incorporated Laponite nanosilicates into the composition.^20^

The morphological characteristics varied across the studies. Six studies reported spherical particles,^19,21,23–25^ while two used nonspherical structures: one with SiO_2_ in the form of spicules derived from marine sponges^17^ and another with needle-like particles due to the presence of Laponite nanosilicates.^20^ Two studies did not specify the particle morphology.^18,22^

The reported particle sizes varied considerably. The smallest particles measured 6.8 nm,^25^ while the largest reached 200 nm.^19^ Other studies reported intermediate values, such as 74 nm^23^ and 45 nm.^24^ The studies that used nonspherical morphologies reported sizes exceeding 30 μm for spicules^17^ and greater than 50 nm for needle-like particles.^20^ Three studies did not report particle size.^18,22,25^

Table 3 summarizes the main physicochemical properties of SiO_2_-containing 3D-printed scaffolds evaluated prior to *in vivo* implantation. Although different analytical techniques were employed across studies (*e*.*g*., SEM, XRD, FTIR), the results are interpreted in terms of the resulting properties, including morphology, porosity, crystallinity, surface chemistry, mechanical performance, degradation behavior, ion release, and wettability. The physicochemical characterization of SiO_2_-containing 3D-printed scaffolds revealed a wide range of properties that are directly relevant to bone regeneration. Morphological and microstructural analyses demonstrated predominantly porous architectures with homogeneous distribution of silica-based phases across most studies. SEM and FESEM observations indicated well-interconnected porous networks, with biosilica spicules embedded within dense alginate matrices,^14^ rough scaffold surfaces due to CS/MSN microsphere incorporation,^15^ and uniformly distributed nSiO_2_ nanoparticles within hydrogel matrices.^16^ In addition, Naga et al.^17^ reported a uniform and porous scaffold surface morphology, confirming the suitability of these materials for tissue ingrowth.

**Table 3:** Physicochemical Properties of SiO_2_-Containing 3D-Printed Scaffolds Evaluated in *In Vivo* Studies

**authors**	**physicochemical characterization methodologies**	**results**
Abdian et al., 2024	FESEM, EDX, XRD, FTIR, compression test and biodegradability	Morphology/Microstructure: Mesoporous SiO_2_–HA particles with average size of 145 ± 1.4 μm.
		Porosity/Pore size: Mesoporous structure.
		Crystallinity/Phase composition: Crystalline hydroxyapatite with reduced amorphous phase due to chitosan incorporation.
		Surface chemistry/Elemental composition: Presence of Ca, P, Si and O; hydroxyl, phosphate, carbonate, silicate and NH_2_ functional groups.
		Mechanical properties: Compressive strength of 25 ± 1.2 MPa.
		Degradation/Ion release/pH: Slow degradation rate.
Sousa et al., 2023	SEM, EDX, FTIR, mass loss, pH evaluation and calcium assay	Morphology/Microstructure: Biosilica spicules embedded in a dense and homogeneous alginate layer.
		Surface chemistry/Elemental composition: Presence of Si, Ca, Al, Fe and trace elements (Zr, Ti, Cu, S, Cr); characteristic alginate and silicate FTIR bands.
		Degradation/Ion release/pH: Mass loss of 30% (day 1) and 46% (day 14); pH decrease from 7.4 to 5.9 with slight recovery; constant Ca release.
Knabe et al. 2023	Porosity, pore size distribution, dissolution behavior and selective ion-release	Morphology/Microstructure: Highly porous scaffold architecture with interconnected pores.
		Porosity/Pore size: ∼ 50% porosity; pore size distribution from ≤ 50 μm to ≥600 μm.
		Degradation/Ion release/pH: Dissolution with release of Si, Mg and Ca ions; selective ion release quantified.
Yuan et al., 2022	SEM and WCA	Morphology/Microstructure: Rough scaffold surface due to incorporation of chitosan/mesoporous silica nanoparticle (CS/MSN) microspheres.
		Surface wettability: Water contact angle of 44.7 ± 1.4°.
Liu et al., 2020	SEM	Morphology/Microstructure: Homogeneous distribution of nSiO_2_ nanoparticles throughout the hydrogel matrix.
Lee et al., 2020	N.D.	N.D.
Ke et al., 2019	XRD and FTIR	Crystallinity/Phase composition: Presence of crystalline tricalcium phosphate (β-TCP) phases.
		Surface chemistry/Elemental composition: FTIR revealed asymmetric P–O vibrations and characteristic β-TCP bands.
Li et al., 2017	N2 adsorption–desorption and XRD	Morphology/Microstructure: Porous scaffold structure with interconnected pores.
		Crystallinity/Phase composition: XRD revealed diffraction peaks indicative of an organized mesoscopic structure.
		Surface chemistry/extural properties: N_2_ adsorption–desorption analysis indicated mesoporosity associated with the silica phase, with an average pore diameter of approximately 6.8 nm.
Naga et al., 2014	SEM, porosity, pore diameter and bending strength and XRD	Morphology/Microstructure: Uniform and porous scaffold surface.
		Porosity/Pore size: Porosity of 84%, 66% and 44% at 1500, 1600, and 1700 °C; mean pore diameter of 83.97 μm.
		Crystallinity/Phase composition: Mullite as the main phase, with alumina and rutile secondary phases.
		Mechanical properties: Flexural strength of 7.1 ± 0.2 MPa.

Porosity-related properties, which play a crucial role in osteoconduction, varied depending on scaffold composition and processing conditions. Average porosity values of approximately 50% were reported by Knabe et al.,^18^ consistent with values observed by Naga et al.^17^ Temperature-dependent porosity changes were also observed, decreasing from 84% at 1500 °C to 66% at 1600 °C and 44% at 1700 °C.^17^ Pore size distribution analysis revealed a heterogeneous structure, with pores ≥600 μm (32.8%), 200–600 μm (18.5%), 50–200 μm (10.7%), and ≤50 μm (38%).^18^ In contrast, Naga et al.^17^ reported a mean pore diameter of approximately 83.97 μm, while Abdian et al.^19^ identified mesoporous SiO_2_–HA particles with average particle sizes of approximately 145 ± 1.4 μm.

Crystallinity and phase composition analyses demonstrated the presence of bioactive calcium phosphate and silica-related crystalline phases. XRD results confirmed crystalline hydroxyapatite in mesoporous SiO_2_–HA scaffolds, accompanied by reduced amorphous peaks attributed to interactions with chitosan.^19^ Ke et al.^20^ reported well-defined crystalline tricalcium phosphate phases, whereas Li et al.^21^ identified diffraction peaks associated with an organized mesoscopic silica framework. In ceramic-based scaffolds, mullite was identified as the dominant crystalline phase (82.1%), with secondary phases of alumina (14.5%) and rutile (3.4%).^21^

Surface chemical composition and functional group analysis further supported the bioactivity of the scaffolds. EDX analyses consistently detected silicon and calcium across studies, with oxygen also identified in SiO_2_–HA composites.^19^ Sousa et al.^14^ additionally reported the presence of elements such as Fe, Zn, Ti, Cu, and Cr. FTIR spectroscopy revealed characteristic hydroxyl group bands between 3200–3430 cm^–1^ and silica-related bands between 800–1022 cm^–1^.^14,19^ Phosphate group vibrations associated with hydroxyapatite were observed at 1058, 600, and 500 cm^–1^, along with carbonate ion peaks at 866 cm^–1^ and NH_2_ absorption bands at 1600 cm^–1^ in chitosan-containing scaffolds.^19^ Alginate-related amide and carbohydrate bands were also detected in composite scaffolds 14, while asymmetric P–O stretching and β-TCP-related bands were identified in TCP-based materials.^16^

Mechanical performance varied according to scaffold composition and structure. Compressive strength values of 25 ± 1.2 MPa were reported for mesoporous SiO_2_–HA scaffolds,^19^ whereas flexural strength values of approximately 7.1 ± 0.2 MPa were observed in ceramic-based scaffolds.^17^ These results indicate that silica-containing scaffolds can achieve mechanical properties compatible with cancellous bone, depending on material design.

Degradation behavior and ion release profiles were evaluated in a limited number of studies but provided important insights into scaffold bioactivity. Abdian et al.^19^ reported a slow degradation rate for mesoporous SiO_2_–HA particles, while Sousa et al.^14^ observed an initial mass loss of approximately 30% within the first day, increasing to 46% by day 14. pH monitoring revealed an initial decrease from 7.4 to 5.9 by day 14, followed by a slight recovery to pH 6.0 at day 21.^14^ Sustained calcium release was consistently observed throughout the experimental period.^14^ In addition, Knabe et al.^18^ reported significant dissolution behavior, with cumulative release of Si, Mg, and Ca ions reaching approximately 120 mg/g after 70 days, and selective ion release values of 0.55 mg/g for Si, 3.6 mg/g for Mg, and 14.5 mg/g for PO_4_^3–^.

Surface wettability was assessed in only one study, which reported a water contact angle of 44.7 ± 1.4°, indicating a moderately hydrophilic surface favorable for cell adhesion.^15^ Finally, only one study did not report physicochemical characterization of the scaffolds prior to implantation,^22^ highlighting the overall emphasis placed on material property evaluation across the included literature.

Table 4 presents the *in vivo* analysis and overall results, highlighting diverse methodologies and outcomes across the studies. Histological evaluations were conducted in seven studies,^17,19–21,23–25^ while bone formation and mineralization were assessed using histomorphometric or radiological analyses in another seven studies^17–22,25^ Additionally, immunohistochemistry was included in four studies,^17,18,23,25^ and specific studies employed SEM/EDS,^21^ RT-PCR,^23^ and macroscopic observations.^23^

**Table 4:** *In Vivo* Analysis and Overall Results

**authors**	***in vivo*** **analyses**	**results**
Abdian et al., 2024	Computed tomography	Mesoporous CS/HA-SiO_2_–HA: significantly higher new bone formation without inflammation
	Histological analysis	Nonimplant group: minimal bone formation in the nonimplant group.
	Immunohistochemical analysis	Bone mineral density: higher and BMP-2 gene expression was markedly high.
Sousa et al., 2023	Histological analysis	BS group: increased BV/TV (%) after 45 days, together with higher Ob.S/BS (%) and N.Ob/T.Ar (mm^2^), but with no differences compared to the control at any time point.
	Histomorphometric analysis	RUNX-2 immunostaining: higher in the BS group at 15 and 45 days, particularly in newly formed bone and around biomaterial particles.
	Immunohistochemistry analysis	OPG immunostaining: increased in these areas, showing a significant increase compared to control at 45 days.
Knabe et al. 2023	Angio-microcomputed tomography analysis	RP group: cells and AVB showed an increase in the surface/volume and thickness of blood vessels from 3 to 6 months.
	Histomorphometric analysis	SSM group: cells and AVB showed reduction in vascular and linear density over time.
	Immunohistochemistry analysis	Bone formation in the RP group reached its peak over time, accompanied by the highest expression of osteogenic markers and enhanced bioactive behavior, which promoted superior vascularization, bone formation, and remodeling.
Yuan et al., 2022	Macroscopic analysis	The SPK@PDA-CS/MSNs group showed superior repair outcomes compared to the SPK@PDA and control groups, with higher scores for blood vessel presence, graft level, and reduced cartilage degeneration.
	Histological analysis	Histological and biochemical analyses revealed increased collagen and GAG content, while immunohistochemistry confirmed elevated collagen II expression.
	Immunohistochemistry analysis	RT-PCR demonstrated significantly higher expression of SOX 9, aggrecan, and collagen II at 1 and 3 months in this group.
	RT-PCR assay	
Liu et al., 2020	Microcomputed tomography analysis	The 2% nSi BPS: superior bone healing, reaching the highest bone volume at 2, 4, 8, and 12 weeks.
	Histological analysis	Microcomputed tomography: confirmed accelerated bone formation
		Fluorescent labeling: revealed new bones, blood vessels and mature structures, highlighting their superior regenerative capacity.
Lee et al., 2020	Histological analysis	After 8 weeks of implantation in nude mice, the Polym and NC bioprinted gels maintained their shape and size, indicating stability. None of the gels allowed vessel growth.
		Both gels presented similar amounts of collagen I and II, with more intense staining of collagen I.
Ke et al., 2019	Histomorphometric analysis	Both samples showed significant osteoid formation, confirming the biocompatibility of TCP.
		After 16 weeks, new mineralized bone (NMB) formation was greater in the Mg–Si-TCP group, indicating greater osteogenesis due to MgO and SiO_2_.
		The 3D printed Mg–Si-TCP scaffolds also had significantly more osteons.
Li et al., 2017	Microcomputed tomography analysis	X-ray, μCT and 3D: better cortical regeneration in MS/CPC/rhBMP-2 scaffolds after 12 weeks, with the highest bone volume. MS/CPC scaffolds outperformed CPCs in repair rate and integrity, while CPC showed the slowest healing.
	Histological analysis	Histological analysis: exhibited the most significant osteogenesis, with new bone filling macropores and blood vessels within them.
		Van Gieson staining: confirmed superior osteogenesis in MS/CPC/rhBMP-2 scaffolds.

Overall, the studies revealed a broad spectrum of results concerning bone formation and tissue maturation. Abdian et al.^19^ reported exceptional new bone formation (90% ± 1.3) in the mesoporous CS/HA-SiO_2_–HA group, contrasting sharply with the minimal bone formation (12% ± 1.5) in the non-implant group. Sousa et al. (2023)^14^ observed effective bone regeneration in the BS group, characterized by increased collagen deposition and active osteoblast presence after 8 weeks. Similarly, Yuan et al. (2022)^15^ demonstrated enhanced repair performance, with improved vascularization, graft integration, and reduced cartilage degeneration. Liu et al. (2020)^16^ highlighted accelerated bone formation, nearly doubling in the initial weeks and achieving 22.67% defect filling by week 8.

Focusing on scaffold stability and biocompatibility, Lee et al. (2020)^22^ found that both Polym and NC bioprinted gels maintained their integrity in nude mice, with histological analysis showing comparable collagen I and II levels. Li et al. (2017)^21^ reinforced these findings by demonstrating robust osteogenesis, with new bone seamlessly integrating with blood vessels. Naga et al. (2014)^17^ confirmed mature bone formation, evidenced by Haversian systems and osteocytes, further validating the scaffold’s effectiveness.

Histomorphometric analyses^17,18,22^ provided valuable insights into bone remodeling and osteogenesis. Sousa et al. (2023) reported increased bone volume (BV/TV %), higher osteoblast surface to bone surface (Ob.S/BS %), and a greater osteoblast count (N.Ob/T.Ar mm^2^) in the BS group, indicating a strong osteogenic potential. Knabe et al.^18^ noted enhanced vascularization and bone remodeling over time benefits of Mg–Si-TCP scaffolds, demonstrating superior mineralized bone formation.

Radiological analyses using computed tomography^18–20,25^ corroborated these findings, offering quantitative insights into bone density and regeneration. Notably, Abdian et al. (2024)^19^ showed increased bone mineral density (0.26 ± 0.01 g/cm^3^m) and elevated BMP-2 gene expression, while Knabe et al. (2023)^18^ observed progressive vascularization and bone formation in the RP group. Liu et al. (2020)^20^ confirmed substantial new bone volume, and Li et al. (2017)^25^ demonstrated the superior performance of MS/CPC/rhBMP-2 scaffolds in promoting cortical regeneration.

Immunohistochemical analyses^17–19,23^ further detailed the expression of osteogenic and inflammatory markers. Sousa et al. (2023)^17^ highlighted higher RUNX-2 and OPG expression, indicating enhanced osteogenic activity, while Yuan et al. (2022)^23^ emphasized increased collagen II expression, underscoring the scaffold’s potential in cartilage repair.

Complementary analytical approaches also supported these conclusions. Naga et al. (2014),^17^ using SEM/EDS, identified a Ca/P ratio of 1.62 in the newly formed bone, demonstrating scaffold biocompatibility. Yuan et al. (2022)^15^ through RT-PCR, revealed significant upregulation of SOX 9, aggrecan, and collagen II, suggesting effective chondrogenic differentiation. Macroscopic observations reinforced these results, showing superior blood vessel presence, graft integration, and reduced cartilage degeneration in the SPK@PDA-CS/MSNs group.

Table 5 presents the assessment of the risk of bias of the studies according to the SYRCLE’s Risk of Bias protocol, classifying the criteria as “Yes”, “No” and “Maybe”.

**Table 5:** Risk of Bias in Individual Studies (SYRCLE’s Rob Toll Criteria)^a^

**SYRCLE’s Risk of Bias Tool**											
	**Selection**			**Performance**		**Detection**		**Attrition**	**Reporting**	**Other**	**Results**
authors	1	2	3	4	5	6	7	8	9	10	
Abdian et al., 2024	Yes	No	Unclear	Yes	Unclear	No	Unclear	Unclear	Yes	Unclear	3
Sousa et al., 2023	Yes	No	Unclear	Yes	Yes	No	Yes	Unclear	Yes	Unclear	5
Knabe et al. 2023	Yes	No	Unclear	Yes	Unclear	Unclear	Unclear	Unclear	Yes	Unclear	3
Yuan et al., 2022	Yes	No	Unclear	Yes	Yes	Yes	Yes	Unclear	Yes	Unclear	6
Liu et al., 2020	Yes	No	Unclear	Yes	Yes	Yes	Unclear	Unclear	Yes	Unclear	5
Lee et al., 2020	Unclear	Unclear	Unclear	No	Unclear	No	Unclear	Unclear	Yes	Unclear	1
Ke et al., 2019	Yes	No	Yes	Yes	Unclear	Yes	Unclear	Unclear	Yes	Unclear	5
Li et al., 2017	Unclear	Unclear	Unclear	Unclear	Unclear	Unclear	Unclear	Unclear	Unclear	Unclear	0
Naga et al., 2014	Unclear	Unclear	Unclear	Unclear	Unclear	Unclear	Unclear	Unclear	Unclear	Unclear	0

a YES answers indicated low risk of bias, NO indicated high risk of bias, and Unclear indicated it was not possible to assign bias.

In terms of selection bias, the studies Abdian et al. (2023),^19^ Sousa et al. (2023),^14^ Knabe et al. (2023)^18^ and Yuan et al. (2022)^15^ presented random allocation of experimental animals, while Liu et al. (2020),^16^ Lee et al. (2020),^22^ Ke et al. (2019)^20^ and Li et al. (2017)^21^ indicated uncertainty in this aspect. Regarding performance bias, Abdian et al. (2024)^19^ Sousa et al. (2023)^14^ and Knabe et al. (2023)^18^ applied blinding of researchers, while Liu et al. (2020),^16^ Lee et al. (2020),^22^ Ke et al. (2019)^20^ and Li et al. (2017)^21^ did not provide sufficient information for this assessment. In detection bias, Abdian et al. (2024),^19^ Sousa et al. (2023)^14^ and Yuan et al. (2022)^15^ ensured blinding in the assessment of outcomes, while Liu et al. (2020),^16^ Lee et al. (2020)^22^ and Li et al. (2017)^21^ presented uncertainty. Attrition bias was identified in Ke et al. (2019),^20^ Liu et al. (2020)^16^ and Li et al. (2017),^21^ who did not clearly report sample losses, while the other studies presented greater clarity in this criterion. In reporting bias, Lee et al. (2020),^22^ Ke et al. (2019)^20^ and Li et al. (2017)^21^ demonstrated uncertainty regarding the full disclosure of results. Other biases were identified in Liu et al. (2020),^16^ Lee et al. (2020)^22^ and Ke et al. (2019),^20^ who presented uncertainty in the standardization of experimental methods. The Naga et al. (2014)^17^ study presented uncertainty in all criteria evaluated. Overall, most studies presented a risk of bias in at least one of the criteria, mainly due to the lack of information on randomization, blinding and transparency in data presentation.

## Discussion

This systematic review aimed to evaluate the use of 3D-printed composite scaffolds containing SiO_2_ in the process of bone regeneration in *in vivo* bone defect models. Most of the studies used rats (Wistar and Sprague–Dawley) and New Zealand rabbits with bone defects in different anatomical regions, such as the calvaria, femur, and radius. The extrusion technique, with alginate-based polymer matrices associated with SiO_2_, was the most common technique used for manufacturing the scaffolds and some studies used other components such as hydroxyapatite, nanocellulose, and collagen. The *in vivo* studies demonstrated that bone defects treated with SiO_2_-containing composite scaffolds presented an accelerated bone matrix deposition, increased collagen production, enhanced vascularization and integration with native tissue, demonstrating the bioactivity of the evaluated scaffolds and their ability to support bone ingrowth.

Beyond the biological outcomes observed, the *in vivo* response to SiO_2_-containing scaffolds is strongly influenced by physicochemical events occurring at the material–tissue interface, particularly local pH modulation. The dissolution of silica and associated ion exchange reactions can alter the microenvironmental pH, a process governed by surface chemistry and surface charge, which in turn regulate protein adsorption and early cell–material interactions.^2,9,23–25^ Classical studies on silica-based bioactive materials, particularly those described by Hench, demonstrated that rapid ion exchange processes may induce transient alkalinization of the surrounding environment, which, when excessive, can result in reduced cell viability and adverse tissue responses rather than promoting regeneration.^26,27^ More recent investigations have confirmed that elevated pH and uncontrolled ionic release from silica-based systems can negatively affect osteoblast proliferation and enhance inflammatory signaling in a concentration- and composition-dependent manner.^23,24,28^ To mitigate these effects, silica-containing scaffolds are frequently combined with alkaline and alkaline-earth ions, such as Na^+^ and Ca^2+^, or incorporated into multicomponent composites, which help buffer local pH, regulate dissolution kinetics, and improve biocompatibility and tissue integration.^20,21,23,29^ These findings highlight that careful compositional design is essential to control pH evolution and accurately interpret SiO_2_-mediated biological responses observed *in vivo*.

Although this review has focused primarily on the biological role of SiO_2_ in bone regeneration, multicomponent glass systems, such as silica-containing bioglasses, have not been explored in depth. This choice does not reflect the exclusion of scaffolds containing multiple oxides, but rather a deliberate conceptual delimitation. Bioglasses represent multicomponent glass materials whose bioactivity emerges from collective dissolution processes and ionic release dynamics that differ fundamentally from those observed in SiO_2_-based systems. The inclusion of these materials would introduce distinct mechanisms of osteogenic stimulation that cannot be directly attributed to silica alone. Thus, these systems were discussed only descriptively and were not included in comparative analyses aimed at isolating SiO_2_-mediated biological responses, ensuring a more precise and mechanistically consistent interpretation of the role of silica in scaffold-mediated bone regeneration.

For the animal models used a notable heterogeneity considering species, sex, age, weight, and bone defect characteristics were used. Rodents, particularly Wistar and Sprague–Dawley rats, were most used due to practical advantages such as low cost and rapid bone healing.^27^ However, these models have limitations in replicating human bone physiology.^28^ New Zealand rabbits, used in three studies, offer a more clinically relevant model because of their thicker cortical bone and closer similarity to human bone microstructure.^29^ This variability reflects the complexity of *in vivo* bone regeneration research and complicates the standardization of outcomes and clinical translation. Bone defect models also varied, with calvarial and femoral critical-size defects (5–8 mm) being most frequent in rats with different implantation periods (2 weeks to 6 weeks). Additionally, different periods of evaluation postimplantation varied from 2 weeks to 6 months, reflecting diverse study objectives but also limiting direct comparisons across results due to the lack of standardization.^30^ It is worthwhile to emphasize that long-term studies are particularly valuable for assessing scaffold integration and remodeling. Taken together, although these findings demonstrated the reliability of the methodologies used, it is necessary to establish more standardized methods for studies involving experimental models of bone defects with the aim of enhancing the quality and comparability of future research.

Compared to hydroxyapatite, β-tricalcium phosphate (β-TCP) exhibits higher solubility and faster degradation kinetics, resulting in increased ionic release and dynamic interaction with bone remodeling. While hydroxyapatite provides long-term structural stability, β-TCP is more rapidly resorbed and replaced by newly formed bone, particularly in critical-sized defects. When combined with SiO_2_, β-TCP-based scaffolds benefit from enhanced surface reactivity and osteogenic signaling, supporting improved bone regeneration outcomes.

Interestingly, SiO_2_ was combined with different polymeric matrices among the included studies, with alginate-based compositions being the most frequently employed. This preference can be attributed to the favorable physicochemical and rheological properties of alginate for extrusion-based 3D printing, including shear-thinning behavior, rapid ionic cross-linking in the presence of divalent cations such as Ca^2+^, and good shape fidelity after printing.^10,11,30,31^ Furthermore, alginate is a biocompatible and biodegradable polysaccharide that enables the homogeneous incorporation of bioactive particles, such as SiO_2_, while preserving scaffold porosity and interconnectivity.^12,32,33^ Its extensive application in bone tissue engineering and bioprinting has been well documented, supporting its suitability as a polymeric matrix for silica-containing scaffolds.^34–36^

SEM and FESEM analysis revealed a porous structure of the scaffold, which are composed by different morphologies, including microparticles, spicules, and microspheres. Porous morphologies, such as those observed in some of the particles, have been associated with better outcomes in terms of bioactivity and bone regeneration due to their ability to promote cell migration and osteogenic differentiation. For example, Knabe et al.^18^ and Li et al. (2017)^25^ observed that scaffold with porosity between 200–600 μm may provide a favorable environment for cell attraction and growth,.^24^ Structural characterization by XRD revealed varying degrees of crystallinity among scaffolds. The presence of crystalline hydroxyapatite and well-defined mesoporous structures, as shown by Abdian et al.^19,25^ and Li et al. (2017),^25^ mimics natural bone matrix behavior and regulates ionic release, favoring bone integration and mineralization.^41^ The incorporation of polymers like alginate and chitosan influenced this crystallinity, reducing amorphous peaks,^19^ which suggests enhanced organization of the hybrid matrix. FTIR analysis supported these findings by identifying functional groups, phosphates, silicates, carbonates, and hydroxyls, key to bioactivity. The presence of NH_2_ groups from chitosan and characteristic alginate bands confirms the interaction between organic and inorganic components, crucial for calcium binding and modulation of surface charge and hydrophilicity.^17,19,36^

The *in vivo* analyses demonstrated enhanced new bone formation,^17–21,23,25^ increased collagen and glycosaminoglycan (GAG) content, in addition to the formation of new blood vessels^23,24^ and glycosaminoglycan (GAG) content,^23^ as well as the formation of new blood vessels.^20,21,25^ These biological events are critical for bone regeneration, as collagen provides the structural framework for mineral deposition, GAGs contribute to matrix hydration and signaling, and vascularization ensures the delivery of nutrients and osteoprogenitor cells to the defect site.^42^ The increases in bone volume, osteoblast number^17^ and remodeling markers highlight the osteoconductive properties of scaffolds and their active role in promoting osteogenic cell recruitment and differentiation.^18–20,22,25,43,44^ This effect likely results from the combined influence of scaffold surface features, porosity, degradation behavior, and bioactive components that stimulate key cellular pathways^45^

Computed tomography analyses further validated the extent and quality of mineralized tissue formation, corroborating the histological findings.^16,21^ Additionally, most studies reported minimal or absent inflammatory responses, suggesting a generally favorable *in vivo* biological profile.

Nevertheless, these findings should be interpreted with caution. Although silica nanoparticles have been extensively investigated for bone tissue engineering due to their bioactivity and osteogenic potential, their biological performance is strongly influenced by physicochemical parameters, including particle size, surface area, porosity, surface chemistry, and concentration. Mesoporous silica nanoparticles have been shown to support cell adhesion and osteogenic differentiation at controlled doses.^25,37^ However, cytotoxic effects have been described in a dose- and size-dependent manner, particularly for smaller particles with higher surface reactivity, which may promote reactive oxygen species (ROS) generation, oxidative stress, and mitochondrial dysfunction.^38–41^ Furthermore, inflammatory responses are closely related to degradation kinetics and surface silanol density, as silica dissolution products can modulate macrophage activation and cytokine release.^42–44^ Although amorphous silica is generally considered safer than crystalline forms, concerns persist regarding long-term accumulation, persistence, and potential immune modulation under high-dose or repeated-exposure conditions.^45,46^

In the studies included in this review, biological safety was primarily inferred from histological evaluation of inflammatory response, tissue integration, and the absence of chronic foreign body reaction. While the lack of chronic inflammation is essential, since persistent inflammation may impair bone healing and promote fibrous encapsulation quantitative assessments of oxidative stress, cytokine profiling, and macrophage polarization were rarely performed.^46^ Moreover, explicit adherence to standardized biocompatibility guidelines, such as ISO 10993, was not consistently reported. This methodological variability represents an important limitation of the current literature and restricts definitive conclusions regarding long-term immunocompatibility and translational safety. Future studies should incorporate standardized and quantitative biological evaluations to better define safe concentration thresholds and immune responses.

Although the studies included in this systematic review primarily focused on local bone regeneration outcomes, the translational safety of silica-containing scaffolds also requires consideration of biodistribution, degradation, and ADME-related aspects.^46^ Importantly, direct pharmacokinetic investigations specifically evaluating 3D-printed silica-based scaffolds remain scarce, and most available evidence derives from nanoparticle-based toxicological models rather than implantable bulk biomaterials.^45,47^ Therefore, the discussion of absorption, distribution, metabolism, and excretion presented here is largely supported by studies involving systemically administered silica nanoparticles.^46^

Following implantation, silica-based scaffolds undergo gradual degradation, predominantly through hydrolytic dissolution of amorphous SiO_2_ into silicic acid (Si­(OH)_4_), a soluble and biologically relevant silicon species.^26^ The dissolution kinetics depend on porosity, surface area, degree of condensation, and incorporation into composite matrices.^26,45^ Controlled degradation is generally considered beneficial in bone tissue engineering, as soluble silica has been associated with stimulation of osteoblastic activity and extracellular matrix mineralization 48. However, excessive or rapid degradation may increase local ionic concentration and potentially enhance systemic exposure.^47^

Evidence derived from nanotoxicology studies indicates that once soluble silica or nanoscale fragments enter systemic circulation, biodistribution is influenced by particle size, surface chemistry, and aggregation state.^46^ Experimental models using intravenously administered amorphous silica nanoparticles have demonstrated accumulation primarily in the liver, spleen, and kidneys, followed by gradual clearance.^46,47^ Renal excretion has been identified as the principal elimination pathway for soluble silicic acid, suggesting relatively efficient systemic clearance under controlled exposure conditions.^47^ It is essential to emphasize, however, that these findings originate from studies investigating dispersed nanoparticles under systemic administration and may not directly reflect the biological behavior of structurally integrated silica within solid scaffolds.^45^

Regarding metabolism, silica does not undergo classical enzymatic biotransformation but is instead eliminated following physicochemical dissolution into silicic acid.^45,49^ Amorphous silica is generally considered less biopersistent and less pathogenic than crystalline polymorphs, which are strongly associated with chronic inflammatory lung disease upon inhalation exposure.^47^ Nevertheless, long-term accumulation, persistence in reticuloendothelial organs, and potential immune modulation remain concerns in high-dose or repeated-exposure scenarios reported in nanoparticle-based studies.^46^

Notably, none of the *in vivo* studies included in this review performed quantitative silicon tracing, systemic biodistribution analysis, or long-term organ toxicity assessment following scaffold implantation. Consequently, while local degradation behavior appears compatible with bone regeneration, comprehensive ADME profiling of silica-containing scaffolds in clinically relevant models remains insufficiently explored.^46^ Future investigations should incorporate silicon quantification in major organs, renal clearance evaluation, long-term histopathological analyses, and standardized degradation kinetics assessments to better define the translational safety window of these biomaterials.

Beyond the descriptive biological outcomes observed *in vivo*, the regenerative potential of SiO_2_-containing scaffolds can be further understood through mechanistic pathways underlying bone repair. Soluble silica species released during scaffold degradation enhance osteogenic differentiation of mesenchymal stem cells, increasing alkaline phosphatase activity and expression of osteogenic markers such as osteocalcin and collagen type I.^48^ Silicate ions also promote angiogenesis by upregulating pro-angiogenic factors including VEGF and endothelial nitric oxide synthase, stimulating endothelial cell proliferation and tube formation *in vitro* and neovascularization *in vivo* ^50,51^ Additionally, strontium-containing silicate-based scaffolds have demonstrated synergistic enhancement of both osteogenic gene expression and angiogenic potential, supporting a dual role in bone regeneration and vascular development.^52,53^

The involvement of silica in endochondral ossification is less extensively studied compared with intramembranous bone formation, but emerging work suggests that tailored silicate microparticles can influence chondrogenesis and cartilage matrix deposition, which are prerequisites for endochondral bone healing. These findings indicate that silica-derived bioactivity may extend across multiple phases of bone repair, although dedicated mechanistic studies on EO induction by SiO_2_-containing composite scaffolds remain limited.

Despite these promising mechanistic insights, translational and regulatory challenges persist. The variability in scaffold composition, degradation profiles, and ion release kinetics complicates reproducibility and regulatory assessment, particularly under standards such as ISO 10993 for biocompatibility and safety evaluation. Furthermore, long-term *in vivo* safety, biodistribution of dissolved silica species, and large-animal validation data are generally scarce, representing important gaps for clinical translation. Consequently, while silica-containing scaffolds demonstrate strong preclinical potential, harmonization of experimental protocols, standardized safety assessment, and alignment with regulatory frameworks remain essential steps toward clinical application.

Beyond safety considerations, scaffolds containing SiO_2_ demonstrated enhanced biological performance, particularly when combined with other bioactive components such as hydroxyapatite,^14^ chitosan,^15,19^ alginates,^16,22^ metal oxides,^17^ and calcium phosphate cements (CPCs).^18,20,21^ These synergistic effects likely result from complementary mechanisms: while SiO_2_ provides bioactive cues that modulate osteogenic signaling pathways, additional components contribute to mechanical stability, controlled degradation, and improved cellular interactions.^29^

Immunohistochemical analyses further supported the osteogenic potential of SiO_2_-containing scaffolds, demonstrating increased expression of key markers, including BMP-2,^19^ RUNX-2,^14^ OPG,^14^ ALP,^18^ OCN,^18^ BSP,^18^ Col I,^15,18^ and GAGs.^15^ Mechanistically, SiO_2_ has been associated with activation of the Wnt/β-catenin and BMP-2 pathways,^27,48^ both of which regulate osteoblast differentiation and extracellular matrix mineralization.^54^ RUNX-2 acts as a master transcription factor in early osteogenesis, activating downstream genes such as ALP, which initiates mineral deposition,^55–58^ and OCN and BSP, which contribute to matrix maturation and stabilization.^55–57,59^ Increased OPG expression observed in SiO_2_-treated groups suggests modulation of bone remodeling dynamics by inhibiting osteoclast-mediated resorption and favoring net bone formation.^20,60^

In addition, PCR analyses of chondrogenic markers such as SOX9, Col II, and aggrecan^61^ indicate that SiO_2_-containing scaffolds may also support cartilage-like tissue formation, an essential step in endochondral ossification during bone repair. GAGs contribute to extracellular matrix hydration and structural organization,^62^ while Col II expression may reflect early cartilaginous or transitional osteogenic phenotypes.^63^ These findings suggest that SiO_2_ acts as a multifunctional bioactive component, influencing both intramembranous and endochondral pathways of bone regeneration.^29,36^

From a structural perspective, 3D printing technology enabled the fabrication of scaffolds with controlled architecture, interconnected porosity, and homogeneous SiO_2_ distribution, closely resembling the native bone microenvironment. Such architectural features are critical for cell migration, vascular ingrowth, extracellular matrix deposition, and subsequent remodeling,^64,65^ likely contributing to the positive *in vivo* outcomes reported across studies.^65^

Despite these promising results, substantial heterogeneity in experimental design, including variations in defect size, anatomical location, animal model, and observation period, limits direct comparison among studies. Although all investigations reported some degree of bone regeneration, the absence of standardized defect models, uniform quantitative histomorphometry, and consistent control groups restricts robust comparative analysis. Harmonization of *in vivo* protocols is necessary to enable future meta-analyses and strengthen translational relevance. Additionally, long-term investigations are essential to determine whether SiO_2_-based scaffolds maintain osteogenic performance and stable integration over extended periods, which ultimately defines their clinical applicability.^27^

## Conclusion

This review analyzed studies on 3D-printed scaffolds containing SiO_2_ for bone regeneration in animal models, considering different species, ages, weights, and types of bone defects. The findings indicate that the incorporation of SiO_2_ into composite scaffolds, particularly those combined with β-tricalcium phosphate (β-TCP), enhances scaffold bioactivity and osteoconductive performance. Rather than acting as an isolated osteoinductive agent, silica plays a synergistic role by modulating surface bioactivity, ionic exchange, and osteogenic signaling pathways, thereby supporting osteoblastic activity, bone formation, and mineralization. In addition, the analyzed studies consistently reported adequate stability, biocompatibility, and vascularization, reinforcing the potential of SiO_2_-containing composite scaffolds for bone repair applications.
